# Trajectories of alcohol consumption prior to the diagnosis of type 2 diabetes: a longitudinal case–cohort study

**DOI:** 10.1093/ije/dyx274

**Published:** 2018-01-12

**Authors:** Craig S Knott, Annie Britton, Steven Bell

**Affiliations:** 1 Research Department of Epidemiology and Public Health, University College London, London, UK; 2 MRC Epidemiology Unit and Centre for Diet and Activity Research (CEDAR), University of Cambridge School of Clinical Medicine, Cambridge Biomedical Campus, Cambridge, UK and; 3 Department of Public Health and Primary Care, Strangeways Research Laboratory, University of Cambridge, Cambridge, UK

**Keywords:** alcohol consumption, trajectories, type 2 diabetes, longitudinal study, Whitehall II

## Abstract

**Background:**

Non-linear associations have been reported between baseline measures of alcohol consumption and type 2 diabetes (T2DM). However, given that drinking varies over the adult life course, we investigated whether differences existed in the longitudinal trajectory of alcohol consumption according to T2DM status.

**Methods:**

For a case–cohort (916 incident cases; 7376 controls) of British civil servants nested within the Whitehall II cohort, the self-reported weekly volume of alcohol consumption was traced backwards from the date of diagnosis or censoring to the beginning of the study, covering a period of up to 28 years. Mean trajectories of alcohol intake were estimated separately by diagnosis status using random-effects models.

**Results:**

Drinking increased linearly among male cases before diagnosis, but declined among male non-cases prior to censoring. At the time of diagnosis or censoring, consumption among those who developed T2DM was 33.4 g/week greater on average. These patterns were not apparent among women. Here, alcohol intake among female cases was consistently below that of non-cases, with the difference in consumption most pronounced around 15 years prior to diagnosis or censoring, at ∼28.0 g/week. Disparities by diagnosis status were attenuated following adjustment for potential confounders, including the frequency of consumption and metabolic factors. Drinking among male and female cases declined following diagnosis.

**Conclusions:**

Differences in the weekly volume of alcohol consumption are reported in the years leading up to diagnosis or censoring. Although male and female cases predominantly consumed alcohol at volumes lower than or equal to those who were not diagnosed, these disparities appear to be largely explained by a range of socio-demographic and lifestyle factors. Where disparities are observed between cases and non-cases, adjusted absolute differences are small in magnitude. The decision to drink alcohol should not be motivated by a perceived benefit to T2DM risk.

**Key Messages**

- Little is known about how alcohol consumption differs throughout adulthood between those that do and do not develop type 2 diabetes mellitus.
- This is the first study to report the mean trajectory of alcohol consumption by diagnosis status across up to 28 years of follow-up.
- Little difference in consumption was apparent between cases and non-cases in the period leading up to diagnosis or censoring after adjustment for confounding factors.
- The decision to drink alcohol should not be motivated by a perceived benefit to type 2 diabetes risk.

## Background

Recent meta-analyses have reported dose–response relationships between the volume of alcohol consumption and the risk of type 2 diabetes mellitus (T2DM).^1^^,^^2^ Specifically, while increased risks of T2DM are evident at high volumes of weekly consumption among both sexes, reductions in risk at moderate volumes appear greatest among^1^ or entirely specific to women.^2^ Aside from the volume consumed, other dimensions of alcohol intake appear to be important modifiers of effect. Though little studied to date,^3^ there are a few indications that the risk of T2DM may be lower at higher frequencies of weekly consumption across both sexes, with both volume and frequency appearing to operate independently of one another.^4^^,^^5^

However, despite indications that drinking behaviours change across the life course,^6^ particularly among heavier drinkers,^7^ with decreases in volume and increases in frequency both observed with increasing age,^6^ studies of alcohol and T2DM risk have predominantly operationalized drinking according to just a single measure of the volume of alcohol consumption.^2^ Little is therefore known about how longitudinal trajectories of alcohol consumption may differ between participants who do and do not develop the condition.

By exploring differences in the trajectory of alcohol consumption according to the diagnosis of T2DM, we can begin to develop a better understanding as to the validity of different hypotheses concerning how increases or decreases in risk reported for different volumes of consumption are likely to be conferred. These include the possibility that risk may accumulate over time as a result of prolonged heavy drinking, or during acute periods of the life course in which sensitivity to the effects of alcohol consumption are most pronounced.^8^ If the risk of T2DM accumulates as a result of chronic heavy drinking, the trajectory of alcohol consumption among those who develop T2DM would be consistently or else predominantly higher on average than among those that do not develop the condition.

In addition, with a growing number of studies linking the onset of ill-health to a subsequent cessation or attenuation of alcohol consumption,^9–11^ it is posited that participants who develop T2DM may exhibit a marked decline in their consumption in line with gradual deterioration in their health status prior to diagnosis.

To examine these hypotheses, this study estimates and compares sex-specific trajectories of the total weekly volume of weekly alcohol consumption according to whether or not participants were diagnosed with T2DM. In addition, to explore changes to drinking behaviour following diagnosis, a further analysis was also undertaken that extended the trajectory of alcohol intake beyond the date of diagnosis. 

## Research design and methods

### The Whitehall II study

The Whitehall II cohort was established in 1985 and enlisted 10 308 (6895 male and 3413 female) civil servants aged 35–55 years who worked in the offices of 20 Whitehall departments.^12^ Data were obtained at each phase via a self-administered questionnaire, with a clinical examination undertaken at every other phase. A fasting plasma glucose test (FPG) was incorporated as part of the clinical examination at phase three (1991–93), with subsequent screening then carried out at phases 5 (1997–99), 7 (2003–04), 9 (2007–09) and 11 (2012–13) alongside self-administered questionnaires. The analytic sample was thus defined as any participant free of T2DM at phase 3 and who participated in at least one subsequent clinical examination such that their event status and follow-up time could be determined. 

### Assessment of alcohol consumption

Alcohol-consumption data were extracted from baseline and all clinical phases noted above. At each phase, participants were asked to report the number of alcoholic drinks they had consumed in the week prior to interview according to ‘measures’ of spirits, ‘glasses’ of wine or ‘pints’ of beer or cider. The study conservatively assumed 8 g of alcohol per measure of spirits or glass of wine and 16 g for each pint of beer or cider. These measurements were then summed to define the total volume of weekly alcohol consumption. Robust standard errors were calculated as the alcohol variable was positively skewed. 

### Assessment of T2DM

Self-reported measures of T2DM were documented at all phases, defined as any self-reported doctor-diagnosis or prescription of anti-diabetic medication. Given that close to one-third of T2DM cases may be missed by self-reports,^13^ subjective measures were supplemented by objective data from phase 3 onwards. Objective cases were identified at each clinical examination following a minimum 5-hour fast, defined according to a FPG test reading ≥7.0 mmol/L in line with the 1998 World Health Organization (WHO) criteria.^14^

### Covariates

BMI was selected as an indicator of adiposity and was calculated using the conventional formula, with measures of height and weight captured at each clinical examination. Ethnicity was self-reported at phases 1 and 5 and coded as ‘White’, ‘South Asian’ or ‘other’. Family history of T2DM (parent or sibling) was self-reported at phases 1 and 2. Information regarding physical activity was ascertained via a 20-item questionnaire that included questions on the frequency and duration of participation in activities including walking and cycling during the 4 weeks preceding each phase. Participants were classified according to WHO physical activity recommendations:^15^ meeting guidelines (≥150 minutes of moderate-intensity exercise per week or ≥ 75 minutes of vigorous-intensity activity); inactive (<60 minutes of moderate physical activity and <60 minutes of vigorous physical activity; below guidelines (anyone not inactive or meeting the WHO guidelines). Smoking data were collected at each phase, with participants categorized according to whether they reported being a current, former or never smoker. Finally, two indicators of socio-economic status were also considered: last known civil service occupational grade (administrative, professional/executive, clerical/support) and employment status (employed, retired, redundant/dismissed/sick/other).

### Statistical analysis

Participants were grouped according to whether or not they developed T2DM over the course of the study. Time was scaled according to the date of diagnosis (for those who developed T2DM) or the final date of participation (for those who were censored), which were each coded as year zero. The self-reported volume of alcohol consumption was then traced backwards to the beginning of the study for each participant. A follow-up time of –15 years thus represents a measure of alcohol consumption collected 15 years prior to diagnosis or censoring, while a decennial change coefficient reported in the results tables refers to the change in alcohol consumption for every 10 years closer to the date of diagnosis or censoring.

## Mean trajectories of alcohol consumption by diagnosis status

Linear trajectories of mean weekly alcohol consumption were estimated for each group using the mixed-effects package (-mixed-) in Stata 13.^16^ Detailed information concerning this process is included within Supplementary Appendices 1 and 2. Briefly, random-effects models were used to allow each participant their own intercept and rate of change per unit of time.

In addition to modelling linear trajectories of alcohol consumption, a range of non-linear slopes were also explored by subjecting the time variable to cubic and quadratic transformation (time^–3^, time^–2^, time^1^, time^2^, time^3^). These transformed variables were then included as predictors of alcohol consumption both singularly and in pairs, permitting a broad range of functional forms. The goodness-of-fit for each resulting model was assessed using the Bayesian information criterion (BIC), which penalizes analyses with a greater number of parameters, thereby helping to avoid any overfitting the underlying data.^17^ An improvement in fit was defined as any reduction in the BIC greater than or equal to a value of 10, relative to a linear random-effects model.^18^

After describing differences in mean alcohol-consumption trajectories by diagnosis status, secondary analyses were undertaken to assess the magnitude by which any disparity between groups could be explained by an unequal distribution of confounding factors. Confounding factors were added incrementally and all models constrained to the same analytical sample as the maximally adjusted model, aiding the comparison of coefficients between models. Four models are reported: Model 1 (unadjusted); Model 2 (as Model 1, plus adjustment for date of birth); Model 3 (as Model 2, plus adjustment for consumption frequency); and Model 4 (as Model 3, plus adjustment for date of birth, BMI, employment status, ethnicity, family history of T2DM, occupational grade, physical activity and smoking status). At each level of adjustment, the statistical significance of differences in intercepts and rates of change between case and non-case participants was assessed via the inclusion of an interaction term between linear time and diagnosis status. Where repeated measures were available, covariates were permitted to vary as a function of time. 

## Probability of transition to non-drinking; sick-quitter effects

When estimating the mean trajectory of alcohol consumption by diagnosis status, changes to the drinking composition of each group are not explicitly defined. For instance, a downward mean trajectory may be indicative of either a gradual overall decrease in the volume of consumption among constituent drinkers or sudden transitions among some participants to complete abstention. To shed light on compositional changes within each mean trajectory, nested logistic regression models were constructed for each group to estimate the probability of transition to non-drinking at each follow-up occasion. This was undertaken using the -xtlogit- package in Stata 13.^16^

Predicted probabilities were then calculated using each logistic regression model and plotted as a function of time. To supplement these plots, a sensitivity analysis was undertaken in which the main linear mixed-effects models were re-run but restricted only to current drinkers (i.e. excluding person-observations where zero consumption was reported). Plotted trajectories within these supplementary models may be considered more robust to the effect of transitions to non-drinking, such as might be attributable to the development of ill-health.

## Changes to alcohol consumption before and after diagnosis

To observe how drinking changes following the development of T2DM, piecewise models were constructed. Using the method described above, separate linear mixed-effects models were constructed according to whether alcohol consumption was reported before or after the documented date of diagnosis. These piecewise models were adjusted for age at the time of diagnosis to account for the possibility that any change following diagnosis may have been confounded in part by advancing age.

## Missing data

Sensitivity analyses were undertaken, which accounted for instance where covariate data were missing due to unit (i.e. a participant did not take part in an entire study phase) or item non-response (i.e. a participant did not answer a given question). Here, an imputation model was created using chained equations.^19^ This predicted the most likely value of each missing datum based upon observed covariates, and thereby operated under the assumption that data were missing at random. Further information concerning the imputation procedure is contained within Supplementary Appendix 3. Results derived using imputed data did not differ markedly from those using complete-case data.

## Results

### Descriptive statistics

**Figure 1.**
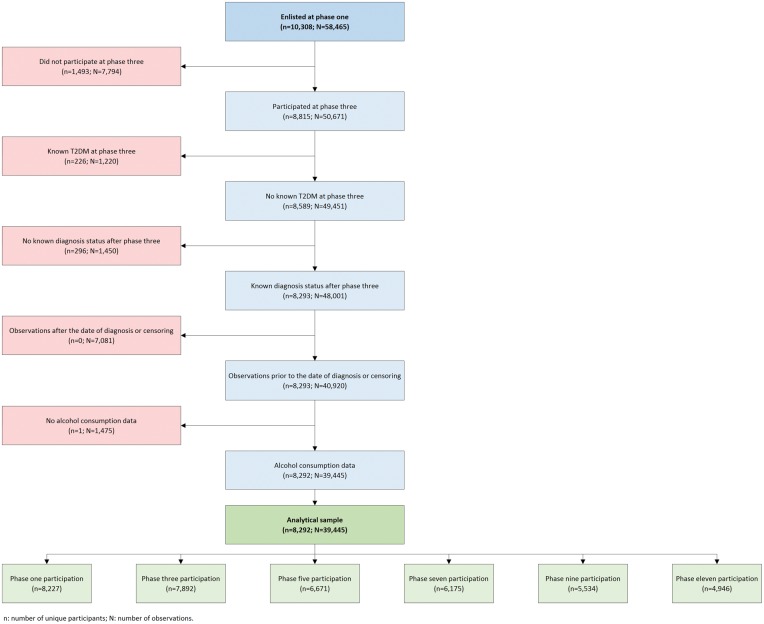
Derivation of the analytical sample.

Of the 10 308 individuals originally enlisted at baseline, a total of 8815 (85.5%) participated at phase 3. Among these, 226 prevalent cases were documented and thus excluded. A total 5723 T2DM-free men and 2570 T2DM-free women had a known incident diagnosis status and follow-up time. After excluding person-observations recorded after the time of diagnosis or censoring, alcohol-consumption data were missing across 3.6% of person-observations. As shown in Figure 1, this left an analytical sample of 5723 men and 2569 women, providing 27 711 and 11 734 observations, respectively. Median follow-up measured 9.9 (IQR 4.1, 25.2) years, with a maximum follow-up of 28.0 years among men and 27.9 years among women. In total, 620 men and 296 women developed T2DM during follow-up.

Participants who developed T2DM had a worse risk profile at baseline than those who did not develop the condition (Table 1), with a greater proportion of such participants being physically inactive, of South Asian ethnicity, in lower occupational grades and having a family history of T2DM, higher BMI and older age. In terms of alcohol consumption, women who developed T2DM reported a lower volume of mean weekly alcohol consumption at baseline.

**Table 1.** Participant characteristics at baseline, stratified by incident T2DM diagnosis during follow-up

	Men			Women		
	T2DM	Censored		T2DM	Censored	
	% (95% CI) *n*	% (95% CI) *n*	*P* for difference^a^	% (95% CI) *n*	% (95% CI) *n*	*P* for difference^a^
**Age**						
Mean years	45.0 (44.6, 45.5)^b^ 620	44.4 (44.2, 44.6)^b^ 5103	0.013	46.7 (46.0, 47.4)^b^ 296	45.3 (45.1, 45.6)^b^ 2274	<0.001
**Alcohol-consumption volume**						
Median g/week	98.8 (89.8, 107.8)^c^ 617	101.5 (98.5, 104.6)^c^ 5067	0.570	29.4 (23.3, 35.5)^c^ 296	46.9 (44.4, 49.4)^c^ 2247	<0.001
**Alcohol-consumption frequency**						
None in past year	4.4 (3.0, 6.3) 27	2.6 (2.2, 3.1) 132	0.028	9.5 (6.6, 13.4) 28	5.7 (4.8, 6.7) 128	<0.001
<1/week	21.8 (18.8, 25.3) 135	19.7 (18.6, 20.8) 1001		51.4 (45.6, 57.0) 152	35.4 (33.4, 37.4) 801	
1–3 times/week	40.3 (36.5, 44.2) 249	43.7 (42.3, 45.0) 2223		28.7 (23.8, 34.2) 85	36.6 (34.6, 38.6) 829	
Daily or almost daily	33.5 (29.9, 37.3) 207	34.1 (32.8, 35.4) 1734		10.5 (7.4, 14.5) 31	22.4 (20.7, 24.1) 507	
**BMI**						
Mean kg/m^2^	26.1 (25.8, 26.3)^b^ 619	24.3 (24.2, 24.4)^b^ 5094	<0.001	28.0 (27.4, 28.6)^b^ 296	24.2 (24.0, 24.3)^b^ 2273	<0.001
**Ethnicity**						
White	84.8 (81.8, 87.5) 526	94.6 (93.9, 95.2) 4815	<0.001	71.2 (65.7, 76.1) 210	89.1 (87.7, 90.3) 2012	<0.001
South Asian	11.8 (9.5, 14.6) 73	3.4 (3.0, 4.0) 175		14.6 (11.0, 19.1) 43	4.6 (3.8, 5.5) 103	
Other^d^	3.4 (2.2, 5.1) 21	2.0 (1.6, 2.4) 101		14.2 (10.7, 18.7) 42	6.4 (5.4, 7.5) 144	
**Family history of T2DM**						
Yes	81.1 (77.8, 84.1) 495	91.2 (90.4, 91.9) 4589	<0.001	68.8 (63.1, 73.9) 198	89.2 (87.8, 90.4) 1989	<0.001
No	18.9 (15.9, 22.2) 115	8.8 (8.1, 9.6) 444		31.3 (26.1, 36.9) 90	10.8 (9.6, 12.2) 242	
**Occupational grade**						
Administrative (top)	35.0 (31.3, 38.9) 217	41.3 (39.9, 42.6) 2106	<0.001	4.1 (2.3, 7.0) 12	14.0 (12.7, 15.5) 319	<0.001
Professional (middle)	54.0 (50.1, 57.9) 335	52.1 (50.7, 53.4) 2657		36.8 (31.5, 42.5) 109	43.0 (41.0, 45.1) 978	
Clerical (bottom)	11.0 (8.7, 13.7) 68	6.7 (6.0, 7.4) 340		59.1 (53.4, 64.6) 175	43.0 (40.9, 45.0) 977	
**Physical activity**^e^						
Inactive	12.4 (10.0, 15.2) 76	8.1 (7.4, 8.9) 410	<0.001	31.9 (26.7, 37.6) 91	22.6 (20.9, 24.4) 504	0.002
Below guidelines	40.1 (36.2, 44.0) 246	37.4 (36.1, 38.8) 1892		34.4 (29.1, 40.1) 98	40.7 (38.7, 42.8) 907	
Met guidelines	47.6 (43.6, 51.5) 292	54.5 (53.1, 55.8) 2753		33.7 (28.4, 39.4) 96	36.6 (34.6, 38.6) 815	
**Smoking**						
Never	42.0 (38.1, 45.9) 258	49.8 (48.5, 51.2) 2525	<0.001	59.0 (53.3, 64.6) 173	54.2 (52.2, 56.3) 1227	0.299
Former	39.3 (35.6, 43.3) 242	36.4 (35.1, 37.7) 1845		22.2 (17.8, 27.3) 65	24.9 (23.2, 26.8) 564	
Current	18.7 (15.8, 22.0) 115	13.8 (12.8, 14.7) 697		18.8 (14.7, 23.7) 55	20.8 (19.2, 22.5) 471	

Sample sizes differed according to item non-response at phase 1. Employment status not listed as all participants were employed at phase 1.

a To explore differences between censored and non-censored groups, one-way ANOVA was used on continuous data, and the chi^2^ test on categorical data.

b Mean and 95% confidence interval (CI).

c Median and 25th and 75th percentiles.

d E.g. Black Caribbean, African and Arabic.

e Meeting guidelines (≥150 minutes of moderate-intensity or ≥ 75 minutes of vigorous-intensity activity per week); inactive (<60 minutes of moderate and <60 minutes of vigorous activity; below guidelines (not inactive or meeting guidelines).

### Trajectories of alcohol consumption up to the date of diagnosis or censoring

**Figure 2.**
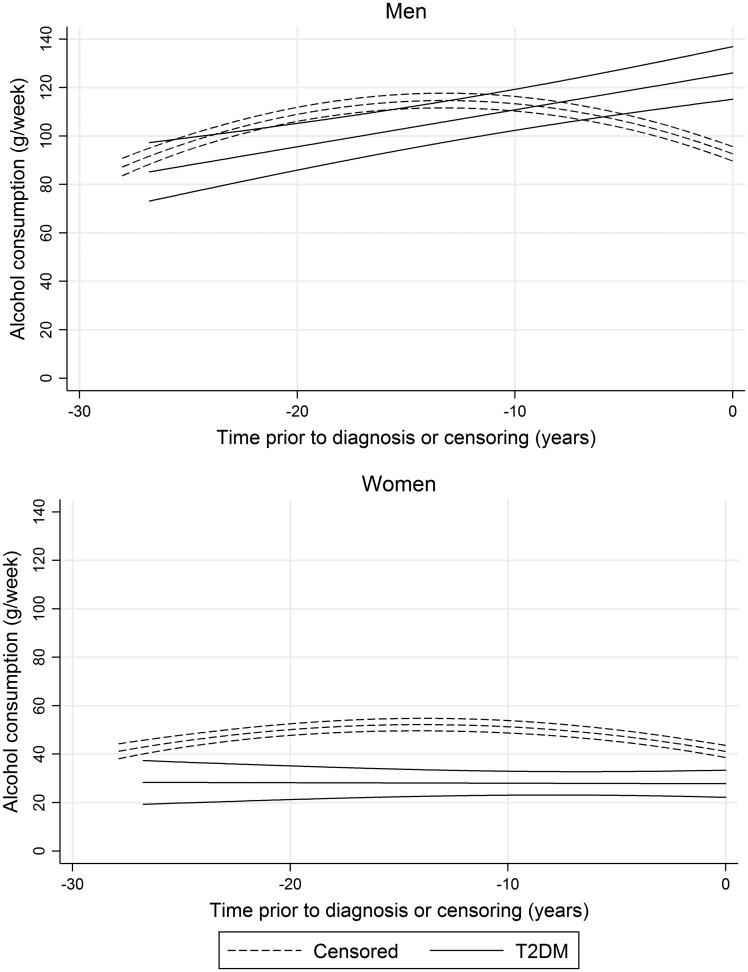
Trajectories of the mean volume of weekly alcohol consumption until the end of follow-up, stratified by sex and T2DM diagnosis.

A range of trajectories were explored, with fit statistics for the corresponding models reported in Supplementary Appendix 4. Trajectories among men and women who developed T2DM were best described as a linear function of time. Conversely, of participants who were censored, a non-linear trajectory provided the best fit of the underlying data (Table 2 and Figure 2).

**Table 2.** Trajectories of the mean volume of weekly alcohol consumption from baseline until the end of follow-up, stratified by sex and T2DM diagnosis

Best-fitting random- effects models	g/week (95% CI)	*p*-value
***Men***		
**T2DM (*n* = 620)**		
Intercept	126.0 (115.2, 136.9)	<0.001
Time^1^	15.2 (9.4, 21.0)	<0.001
**Censored (*n*=5103)**		
Intercept	92.6 (89.6, 95.6)	<0.001
Time^1^	–33.2 (–36.6, –29.8)	<0.001
Time^2^	–1.3 (–1.4, –1.1)	<0.001
***Women***		
**T2DM (*n*=296)**		
Intercept	27.8 (22.2, 33.4)	<0.001
Time^1^	–0.2 (–4.1, 3.7)	0.919
**Censored (*n*=2273)**		
Intercept	41.1 (38.6, 43.6)	<0.001
Time^1^	–15.9 (–19.0, –12.7)	<0.001
Time^2^	–0.6 (–0.7, –0.4)	<0.001

Intercept coefficients refer to the average volume of weekly alcohol consumption at the time of diagnosis or censoring. Time coefficients refer to the change in the average volume of weekly alcohol consumption per 10 years closer to diagnosis or censoring. Superscript numbers for time refer to power terms.

At 30 years prior to diagnosis or censoring, the mean volume of weekly alcohol consumption was estimated to be roughly equivalent among cases and non-cases in men, at around 80.0 g/week. However, by the time of diagnosis or censoring, the mean volume of weekly alcohol consumption among men who did not develop T2DM was lower than among men who developed the condition, at 92.6 g/week and 126.0 g/week, respectively. This equated to a difference of 33.4 g/week, or around 1.8 pints of 4.0% ABV lager.^20^

Among women, consumption remained consistently higher among those that did not develop T2DM. Differences in the mean volume of alcohol consumption by T2DM diagnosis were greatest at 15 years prior to the time of diagnosis or censoring, at around 28.0 g/week. Differences were most acute at both the beginning and end of the follow-up period, equal to 13.3 g/week at the time of event or censoring, or around 0.7 pints of 4.0% ABV lager.^20^

### Multivariable-adjusted trajectories of alcohol consumption up to the date of diagnosis or censoring

As indicated in Table 1, T2DM risk factors were differentially distributed between cases and non-cases. To test the effect of confounder adjustment upon disparities in the trajectory of consumption by T2DM status, models were incrementally adjusted for these covariates.

Results are reported in Table 3 and displayed in Supplementary Appendix 5. Differences in consumption at the time of diagnosis or censoring were attenuated among men from 20.4 g/week (95% CI 8.9, 32.0) to 12.5 g/week (95% CI 3.2, 21.8) following adjustment for confounding factors, with disparity in the mean rate of change halved in magnitude. Among women, differences in consumption at the end of follow-up were also markedly reduced, falling from 20.4 g/week (95% CI 14.0, 26.7) to 7.8 g/week (95% CI 3.1, 12.4). There was no shift in the mean rate of change over time.

**Table 3.** Unadjusted and adjusted linear trajectories of the mean volume of weekly alcohol consumption from baseline until the end of follow-up, stratified by sex with an interaction by T2DM diagnosis

	Model 1		Model 2		Model 3		Model 4	
Linear random-effects models	g/week (95% CI)	*p*-value	g/week (95% CI)	*p*-value	g/week (95% CI)	*p*-value	g/week (95% CI)	*p*-value
***Men***								
**Consumption volume**								
Intercept	104.5 (101.3, 107.7)	<0.001	84.9 (79.2, 90.7)	<0.001	132.6 (127.7, 137.5)	<0.001	110.4 (104.6, 116.2)	<0.001
Change per 10 years closer to diagnosis or censoring	–0.4 (–1.7, 0.9)	0.527	–0.4 (–1.6, 0.9)	0.551	–4.2 (–5.4, –3.1)	<0.001	–7.8 (–9.1, –6.5)	<0.001
**Difference in consumption at the time of diagnosis or censoring**								
Censoring	Reference		Reference		Reference		Reference	
T2DM	20.4 (8.9, 32.0)	0.001	21.5 (10.0, 33.0)	<0.001	21.0 (11.6, 30.5)	<0.001	12.5 (3.2, 21.8)	0.008
**Difference in the rate of change by diagnosis or censoring**								
Censoring	Reference		Reference		Reference		Reference	
T2DM	15.3 (9.1, 21.6)	<0.001	15.4 (9.2, 21.6)	<0.001	10.9 (5.2, 16.5)	<0.001	8.0 (2.4, 13.6)	0.005
**Alcohol-consumption frequency**								
None in past year	–		–		–143.8 (–148.6, –138.9)	<0.001	–137.9 (–143.1, –132.7)	<0.001
<1/week	–		–		–115.7 (–119.1, –112.3)	<0.001	–113.0 (–116.5, –109.6)	<0.001
1–3 times/week	–		–		–77.3 (–80.3, –74.3)	<0.001	–76.1 (–79.1, –73.1)	<0.001
Daily or almost daily	–		–		Reference		Reference	
*Log-likelihood*	*–142 192*		*–142 167*		*–139 920*		*–139 692*	
*Bayesian information criterion*	*284 464*		*284 426*		*279 960*		*279 626*	
*Sample size*	*5625*		*5625*		*5625*		*5625*	
***Women***								
**Consumption volume**								
Intercept	46.95 (44.29, 49.62)	<0.001	34.82 (30.27, 39.4)	<0.001	92.4 (87.7, 97.0)	<0.001	91.4 (85.7, 97.2)	<0.001
Change per 10 years closer to diagnosis or censoring	–1.0 (–2.1, 0.0)	0.059	–1.0 (–2.0, 0.0)	0.06	–1.2 (–2.2, –0.3)	0.011	–1.5 (–2.5, –0.5)	0.002
**Difference in consumption at the time of diagnosis or censoring**								
Censoring	Reference		Reference				Reference	
T2DM	–20.4 (–26.7, –14.0)	<0.001	–18.9 (–25.3, –12.6)	<0.001	–7.7 (–12.0, –3.4)	<0.001	–7.8 (–12.4, –3.1)	0.001
**Difference in the rate of change by diagnosis or censoring**								
Censoring	Reference		Reference				Reference	
T2DM	0.9 (–3.0, 4.9)	0.639	1.1 (–2.9, 5.0)	0.596	–0.5 (–3.7, 2.7)	0.750	–1.1 (–4.4, 2.1)	0.495
**Alcohol-consumption frequency**								
None in past year	–		–		–93.5 (–97.5, –89.6)	<0.001	–88.8 (–93.1, –84.5)	<0.001
<1/week	–		–		–82.3 (–86.0, –78.7)	<0.001	–79.9 (–83.7, –76.1)	<0.001
1–3 times/week	–		–		–55.1 (–58.6, –51.6)	<0.001	–53.9 (–57.4, –50.4)	<0.001
Daily or almost daily	–		–		Reference		Reference	
*Log-likelihood*	*–52 344*		*–52 327*		*–50 918*		*–50 854*	
*Bayesian information criterion*	*104 762*		*104 738*		*101 947*		*101 929*	
*Sample size*	*2492*		*2492*		*2492*		*2492*	

Model 1: unadjusted; Model 2: as Model 1, plus adjustment for date of birth; Model 3: as Model 2, plus adjustment for consumption frequency; Model 4: as Model 3, plus adjustment for BMI, employment status, ethnicity, family history of T2DM, occupational grade, physical activity and smoking status.

### Transitions to non-drinking

**Figure 3.**
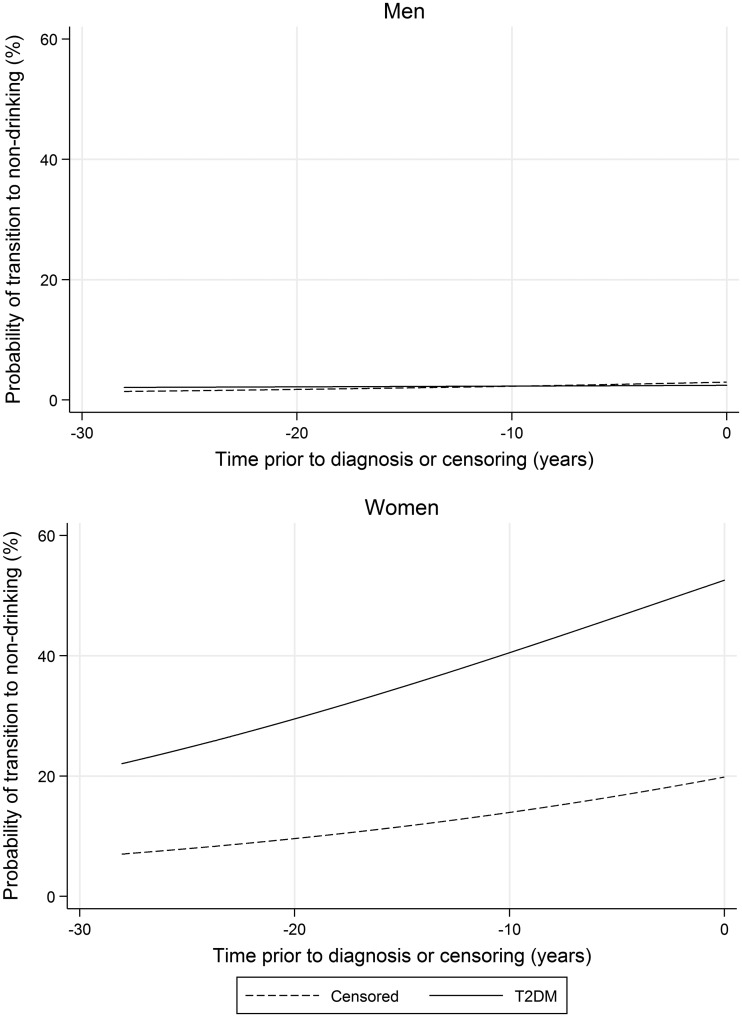
Probability of transition to non-drinking, stratified by sex and diagnosis status.

As shown in Figure 3, the probability of transition to abstention was low among men regardless of diagnosis status (*p* = 0.934), with no difference between the two groups in the probability of transition as a function of time (*p* for interaction = 0.123). Among women, the probability of transition was consistently higher among female cases than non-cases (*p*≤0.001), with no difference by diagnosis status as a function of time (*p* for interaction=0.630). When person-observations with zero consumption were excluded from the models reported in Table 3, differences by diagnosis status were little changed among both sexes (Supplementary Appendix 6).

### Trajectories of alcohol consumption beyond the date of diagnosis

**Figure 4.**
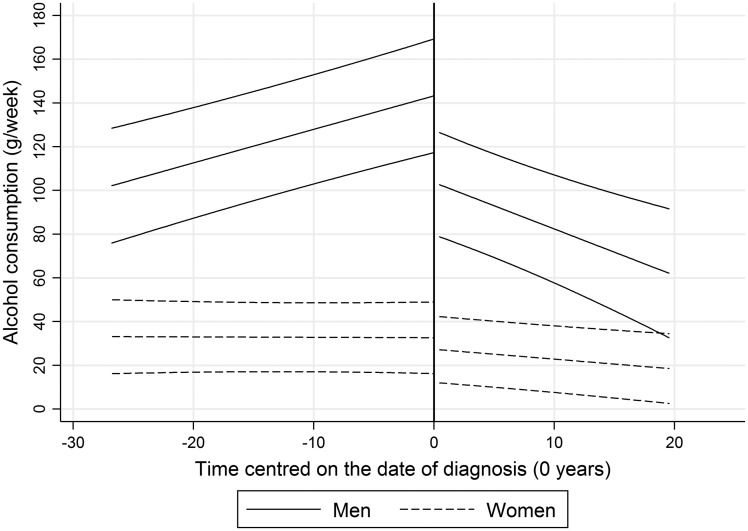
Piecewise age-adjusted trajectories of the mean volume of weekly alcohol consumption before and after the date of diagnosis, stratified by sex.

Of the 620 men and 296 women who developed T2DM over the course of the study, 552 and 267 participants provided alcohol-consumption data after their date of diagnosis. Trajectories of alcohol consumption were estimated based upon a total 3262 person-observations among men and 1513 person-observations among women. Goodness-of-fit statistics for each piecewise model are reported in Supplementary Appendix 7. Linear trajectories provided the best fit of the underlying data and are reported in Table 4 and Figure 4. Significant reductions in consumption were evident among both sexes following diagnosis, equal to a mean 21.2 g/week per decade among men and 4.5 g/week per decade among women.

**Table 4.** Piecewise age-adjusted trajectories of the mean weekly volume of alcohol consumption before and after the date of diagnosis, stratified by sex

	Men		Women	
Piecewise models	g/week (95% CI)	*p*-value	g/week (95% CI)	*p*-value
**Before the date of diagnosis**				
Intercept^a^	143.3 (117.3, 169.2)	<0.001	32.6 (16.2, 49.0)	<0.001
Time^1^^b^	15.3 (9.5, 21.1)	<0.001	–0.2 (–4.1, 3.7)	0.925
**After the date of diagnosis**				
Intercept^c^	103.6 (79.7, 127.5)	<0.001	27.3 (12.2, 42.5)	<0.001
Time^1^^b^	–21.2 (–32.2, –10.3)	<0.001	–4.5 (–7.9, –1.2)	0.008

a The average volume of weekly alcohol consumption at the time of diagnosis.

b The linear change in the average volume of weekly alcohol consumption per 10 years of follow-up.

c The average volume of weekly alcohol consumption at the first phase of measurement following diagnosis. Models are adjusted for date of birth.

## Conclusions

Recent meta-analyses have reported an increased risk of T2DM among both sexes at higher volumes of average daily^1^ or weekly^2^ alcohol consumption. Relative to individuals who did not develop T2DM, it was thus hypothesized that those diagnosed with T2DM would exhibit a consistently higher volume of alcohol consumption prior to diagnosis. To test this hypothesis, random-effects models were constructed to examine differences in trajectories of alcohol consumption by T2DM diagnosis.

Our findings do not support the supposition that the risk of T2DM may accumulate as a consequence of prolonged exposure to heightened volumes of alcohol. Women diagnosed with T2DM consistently consumed alcohol at volumes that were lower on average than those who were censored, while men who developed the condition consumed alcohol at lower or equivalent volumes than non-cases until just a few years prior to the end of the follow-up period. Although there is therefore a possibility that an increased risk of T2DM may be conferred among men as a consequence of acute heavy consumption later in the life course—a period during which sensitivity to the deleterious effects of higher alcohol-consumption volumes may be most pronounced^21–25^—the adjusted difference in consumption at this time was just 12.5 g/week, or around two-thirds of a 4.0% ABV pint of lager per week.^20^

Consumption among female cases and non-cases was consistently within the range of intake associated with reductions in the risk of T2DM.^2^ At least two reasons for this apparent contradiction are possible. First, the mean trajectory for women who developed T2DM may have comprised not primarily of persistent low-volume and therefore lower-risk drinkers, but of higher-risk sick quitters or former heavy drinkers who had attenuated their drinking owing to poor health.^11^^,^^26^^,^^27^ This was supported by the higher probability of transition to non-drinking among female cases than non-cases (Figure 3), their worse metabolic risk profile at baseline than those who were censored and attenuated reductions in T2DM risk as reported elsewhere when former drinkers are excluded from non-drinking reference categories.^2^^,^^28^^,^^29^ However, despite a higher probability of transition to non-drinking among female cases within the cohort, the exclusion of person-observations where zero consumption was reported resulted in an attenuation of differences by diagnosis status (Supplementary Appendix 6). A second possibility was that female reductions in T2DM risk associated with lower volumes of alcohol^2^ may have been a statistical artefact attributable to poor confounder adjustment, with 39% of selected studies having only provided unadjusted or age-adjusted risk estimates.^2^ As per results from recent dose–response meta-analyses concerning alcohol consumption and T2DM risk,^1^^,^^2^ there were clear sex-specific disparities in the trajectories of alcohol consumption prior to diagnosis. Understanding the determinants of these differences is important and merits further detailed investigation.

Drinking among male and female non-cases declined on average during the decade preceding censoring. An analysis of Whitehall II participants aged 61–85 years at phase 11 reported a broad range of reasons for participants reducing their consumption into later life.^30^ Of the 40% who attenuated their intake over the preceding decade, 21% of men and 22% of women did so in response to illness or pharmacological contraindication, and 45% of men and 34% of women as a health precaution. It is possible that the downward trajectory among non-cases was a combination of such factors. Whatever the predominant motivation, the lack of a similar downward trajectory among men who developed T2DM conflicts with the hypothesis that declining health prior to the onset of T2DM would elicit a reduction in consumption during a period preceding diagnosis. Instead, when the trajectory was extended among cases, reductions were apparent only after the date of diagnosis. It is unclear whether this decline was a self-motivated response to a deterioration in health or a reaction to formal medical advice.

### Study strengths and limitations

This is the first study to describe the trajectory of alcohol consumption across the adult life course prior to T2DM diagnosis. Analyses of the Whitehall II cohort benefitted from six phases of observation, objective ascertainment of T2DM cases and good coverage of the adult life course. In addition, despite representing a geographically concentrated and occupationally narrow cohort, aetiological associations within Whitehall II are consistent with those reported from studies of general-population samples.^31^ Mean consumption reported by Whitehall II participants is harmonious with nationally representative, UK-based cohorts.^6^ However, analyses of Whitehall II data were dependent upon self-reported measures of alcohol consumption. Consequentially, plotted trajectories of mean exposure risked being subject to some degree of reporting or recall bias.

A further limitation concerned the restriction of the non-linear models to just two polynomial terms, which constrained slopes to just one turning point. This constraint risked the plotted trajectories being simplistic if multimodal curves were present within the underlying data. However, based on results elsewhere,^6^ multimodal trajectories appear unlikely. Moreover, while the iterative addition of further polynomial terms can improve the specification of statistical models, doing so comes at the cost of diminished external validity.

Although this study included adjustment for a broad range of demographic and lifestyle factors, two limitations are noted. First, owing to the nutritional and metabolic effects of alcohol and alcoholic drinks, there is a possibility that BMI may operate on the causal pathway between drinking and T2DM. Unfortunately, research in this area is conflicting. For instance, while Mendelian randomization studies indicate a positive relationship between alcohol consumption and markers of adiposity,^32^ it remains inconclusive whether alcohol-derived calories are sufficiently additive to meal-derived calories as to increase the risk of metabolic disease in a clinically meaningful way.^33^^,^^34^ In addition, at least one GWAS analysis indicates a negative association between alcohol consumption and anthropometric measures,^35^ the longest-running alcohol-feeding experiment to date shows no difference in weight change between exposed and unexposed groups,^36^ and at least one randomized–controlled trial reports that alcohol dosing affects glycaemic traits similarly irrespective of body mass.^37^ Given that the aim of this study was not to estimate the association between a given level of consumption and T2DM risk, but rather to predict changes in alcohol consumption over time, an a priori decision was made to treat adiposity as a confounding factor. For reference, models are included in Supplementary Appendix 8 that report multi-variable-adjusted linear trajectories of alcohol consumption with and without the inclusion of BMI. Coefficients vary little, suggesting that, if BMI is a mediator, its role in this particular analysis is marginal.

A second issue concerned the possibility that non-alcohol-derived calories may represent a source of residual confounding. Although a food-frequency questionnaire was administered at each phase, dietary composition data (e.g. fats, carbohydrates and fibre) were only derived in Whitehall II for phases 3, 5 and 7, meaning that the inclusion of diet-related variables would have necessitated a substantial reduction in the analytical sample.

Aside from the issue of confounding, reverse causality was possible among participants with fewer than three person-observations. Among such individuals, the precise ordering of changes to alcohol consumption and diabetes status between two observations are unknown, with each being documented concurrently at each phase of observation. Of the 916 individuals known to have developed T2DM over the period of follow-up, 222 (24.2%) provided fewer than three person-observations, indicating that close to one-quarter of known cases may have induced changes to the alcohol-consumption trajectory. To assess this further, a sensitivity analysis was undertaken whereby the maximally adjusted models reported in Table 3 were restricted to participants with at least three person-observations. Although not directly comparable due to their differing samples, negligible difference in coefficients is evident beyond an expected reduction in precision (Supplementary Appendix 9), suggesting that our findings are unlikely to be entirely due to reverse causation.

Finally, missing data were such that analyses may have been applied to a healthier sub-sample of the source population, impairing generalisability and potentially underestimated the incidence of T2DM cases. For instance, Whitehall II participants with unit or item non-response at any phase during the period of follow-up exhibited a worse metabolic profile at baseline than those with complete data (Supplementary Appendices 10 and 11). However, results based upon analyses of an imputed dataset were comparable.

### Summary

Our findings do not support the notion that the harms or alleged benefits of alcohol consumption for T2DM risk accumulate over time. Where differences were apparent by diagnosis status, these were markedly attenuated following adjustment for T2DM risk factors. Based upon results from recent dose–response meta-analyses,^1^^,^^2^ differences were of magnitudes that do not appear to be clinically important. Given the absence of evidence indicating that mean consumption was markedly higher among those diagnosed with T2DM, the decision to take up drinking should not be motivated by a perceived benefit to T2DM risk. Despite suggestions that moderate drinking may be advantageous for health,^38^ such advice seems premature in this context. Indeed, taking a population perspective, some academics recommend that drinking guidelines explicitly discourage alcohol consumption for perceived health benefits.^39^ This standpoint seems especially prudent given research which indicates that adults who believe alcohol is beneficial for their health drink alcohol in greater quantities than those who do not or are unsure.^40^ Further research is now required to better understand why trajectories of alcohol consumption differ so markedly between men and women, with more detailed analyses into how trajectories may differ according to alternative dimensions of drinking behaviour.

## Supplementary Data

The appendix is available as Supplementary data at *IJE* online.

## Funding

This work was supported by the European Research Council (ERC-StG-2012–309337_AlcoholLifecourse) and a Medical Research Council/Alcohol Research UK grant (MR/MM006638/1). The funders had no role in study design, data collection or analysis, decision to publish or preparation of the manuscript. The views expressed are those of the authors and not necessarily those of the funders. The corresponding author had full access to all the data in the study and had final responsibility for the decision to submit for publication.

## Supplementary Material

Supplementary Data
