# Supplementary material for: Trajectories of alcohol consumption prior to the diagnosis of type 2 diabetes: a longitudinal case–cohort study
Source: Int J Epidemiol. 2018 Jan 12;47(3):953–65. doi: 10.1093/ije/dyx274 (PMC6005149; doi:10.1093/ije/dyx274)
Supplement: Supplementary Data [file dyx274_ije-2017-01-0118-file002.docx]

**Appendices**

**Appendix 1 Calculating trajectories of alcohol consumption using mixed effects models**

Although trajectories of alcohol consumption could have been calculated using a standard linear regression model, this approach treats repeated measures as a series of unique and independent data points, leading to an overestimation of precision. Accordingly, consumption trajectories were calculated using mixed effects models, which inflate standard errors proportionate to the magnitude of correlation between repeated measures within individuals.

The resulting random intercept model is expressed in general terms per Formula 1, with the subscript *i* denoting the participant and the subscript *j* denoting the repeated measure. The “true” predicted value of weekly alcohol consumption at the time of the *j^th^* measurement for the *i*^th^ participant is therefore calculated as the estimated mean intercept (β_0_) plus a random effect denoting the participant’s predicted deviation from the mean intercept value (b_0i_), with a fixed slope (β_1_(t_ij_)) that equates to the estimated mean rate of change in alcohol consumption per unit of time. Random error is denoted by ε_ij_, representing any remaining variation in alcohol consumption not explained by the model.

y_i_(t_ij_)=(β_0_+b_0i_)+(β_1_)t_ij_+ε_ij_

**Formula 1 Calculation of a linear random intercepts model**

The random intercept model is illustrated in Appendix 2a and assumes that all participants share the same rate of change in alcohol consumption over time, which is unlikely to reflect reality**.** To capture any underlying differences in slopes between participants, the random intercepts model is expanded to allow random slopes (Formula 2). The resulting model allows each participant to exhibit their own rate of change in alcohol consumption per unit of time by adding a random effects term to the mean slope coefficient. This random effects term denotes the predicted deviation of each participant-specific slope from the mean slope (b_1i_(t_ij_)). Finally, an unstructured covariance matrix is specified, which allows within-participant covariance between the intercept and repeated measures to take any form. This model is illustrated in Appendix 2b.

y_i_(t_ij_)=(β_0_+b_0i_)+(β_1_+b_1i_)t_ij_+ε_ij_

**Formula 2 Calculation of a linear random intercepts and random slopes model**

**Appendix 2 An illustration of random intercept and random slopes models**


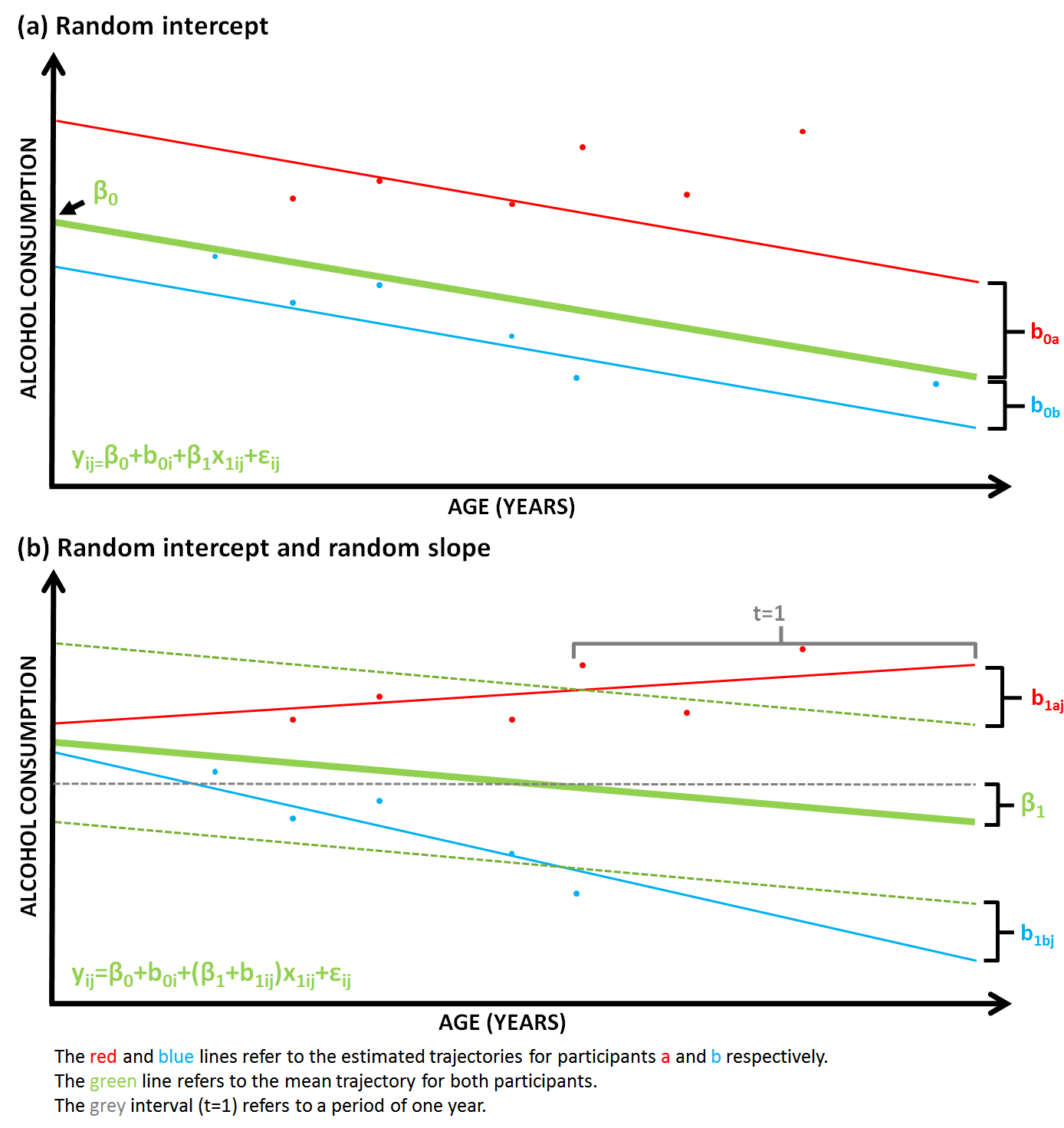


**Appendix 3 Construction of the chained equations multiple imputation model**

Variables with missing data were imputed iteratively via a sequence of regression models that predicted missing data conditional on all other observed variables within the imputation model.

First, for each variable with missing data, the most appropriate corresponding regression model was identified. For instance, in the case of binary data, a logistic regression model was chosen. Where continuous variables were skewed, observed data were log transformed prior to imputation, then converted back to their original scale post-imputation. Observed variables were selected *a priori* on the grounds that they were considered likely to be correlated with the probability of missingness. Aside from the substantive analytical variables of interest, including confounding factors, these were: the General Health Questionnaire,^[[1]](#endnote-1)^ the CAGE questionnaire^[[2]](#endnote-2)^ and Mini Mental State Examination^[[3]](#endnote-3)^ scores; self-rated general health (‘very good or excellent’, ‘good’, ‘fair or poor’); the amount of time participants reported being ‘worn out’ in the four weeks prior to each wave ('all of the time', 'most of the time', 'a good bit of the time', 'some of the time', 'a little of the time', 'none of the time'); whether family obligations reduced a participant’s time available for relaxation ('not at all', 'to some extent', ' a great deal', 'not applicable [e.g. no family obligations]’). Diagnosis status was operationalised via three variables: the number of new cases captured at each wave of measurement, a continuous variable equal to the time to diagnosis or censoring for each participant, and the cumulative baseline hazard function. Failure to include such variables risked biasing toward the null any association between covariates and T2DM when using imputed data.^[[4]](#endnote-4)^,^[[5]](#endnote-5)^

The imputation procedure then began by populating all missing data with random values to permit the inclusion of missing data points within each regression model. Starting with the variable for which the proportion of missing data was lowest (x_1_), observed data on that variable were regressed against all other selected covariates (x_2_,...,x_p_). Missing values on x_1_ were then replaced by the most likely predicted values_,_ given x_2_,...,x_p_. Once missing values on x_1_ were predicted, the imputation procedure moved to the variable with the next lowest proportion of missing data (x_2_). Observed values of x_2_ were then regressed on all other covariates (x_1_,x_3_,…,x_p_), with x_1_ operationalised to include all data predicted at the preceding stage. This process continued iteratively until missing values had been imputed for all variables with missing data, representing one complete iteration of the imputation procedure.

At the start of the second iteration, missing data were populated with predictions from the previous iteration as opposed to random noise. The calculation of new iterations then continued until the predicted values were consistent between iterations. In such a circumstance, variation in predicted values between iterations appeared random and the data were said to have converged. This was established visually using trace plots, and was achieved for all variables following 40 iterations.

At the point of convergence, predicted data were stored in a dataset as one complete imputation. To capture the degree of uncertainty surrounding these predicted values, 50 imputations were run – a value equal to at least the total proportion of participants without complete-case data.^[[6]](#endnote-6)^

The imputation procedure was completed while data were stored in their wide format. This modelled each wave of a given variable as a separate covariate, allowing for the modelling of missing data as a function of observations at all times that preceded and followed the wave of unit or item non-response. Data were imputed using Stata 13 and the -mi- package. The *augment* option was selected to bypass any circumstance in which perfect prediction between variables may have occurred, by adding random observations with very low weights to the included variables.

Imputed estimates were examined to identify any incongruous values, such as negative alcohol consumption. Where invalid values were identified, the imputation model was modified and re-run, such as by utilising a truncated regression model, which restricted the lower limit of predicted alcohol consumption to a value of zero.

**Appendix 4 Goodness of fit statistics for linear and non-linear trajectories of mean weekly alcohol consumption, stratified by sex and T2DM diagnosis.**

|  | **T2DM** | |  | **No T2DM** | |
| --- | --- | --- | --- | --- | --- |
| **Linear and non-linear functions of follow-up time (years)** | **Log-likelihood** | **^a^BIC** |  | **Log-likelihood** | **^a^BIC** |
| **Men** |  |  |  |  |  |
| time^-2^ | -12665 | 25361 |  | -149882 | 299804 |
| time^-1^ | -12665 | 25361 |  | -149866 | 299772 |
| time^1^ | -12648 | 25327 |  | -149877 | 299794 |
| time^2^ | -12647 | 25324 |  | -149882 | 299805 |
| time^3^ | -12649 | 25329 |  | -149876 | 299792 |
| time^-2^+time^-1^ | -12665 | 25368 |  | -149813 | 299678 |
| time^-2^+time | -12648 | 25334 |  | -149875 | 299801 |
| time^-2^+time^2^ | -12647 | 25332 |  | -149880 | 299812 |
| time^-2^+time^3^ | -12649 | 25336 |  | -149874 | 299799 |
| time^-1^+time | -12648 | 25334 |  | -149862 | 299774 |
| time^-1^+time^2^ | -12647 | 25332 |  | -149864 | 299778 |
| time^-1^+time^3^ | -12649 | 25336 |  | -149857 | 299764 |
| time+time^2^ | -12647 | 25332 |  | -149709 | 299468 |
| time+time^3^ | -12647 | 25333 |  | -149730 | 299510 |
| time^2^+time^3^ | -12646 | 25331 |  | -149779 | 299609 |
|  |  |  |  |  |  |
| **Women** |  |  |  |  |  |
| time^-2^ | -4758 | 9543 |  | -57154 | 114345 |
| time^-1^ | -4758 | 9543 |  | -57153 | 114343 |
| time^1^ | -4759 | 9545 |  | -57152 | 114342 |
| time^2^ | -4759 | 9545 |  | -57159 | 114355 |
| time^3^ | -4759 | 9545 |  | -57159 | 114355 |
| time^-2^+time^-1^ | -4758 | 9549 |  | -57152 | 114351 |
| time^-2^+time | -4758 | 9550 |  | -57147 | 114340 |
| time^-2^+time^2^ | -4758 | 9550 |  | -57154 | 114354 |
| time^-2^+time^3^ | -4758 | 9550 |  | -57154 | 114354 |
| time^-1^+time | -4758 | 9549 |  | -57146 | 114339 |
| time^-1^+time^2^ | -4758 | 9549 |  | -57152 | 114351 |
| time^-1^+time^3^ | -4758 | 9549 |  | -57153 | 114352 |
| time+time^2^ | -4759 | 9552 |  | -57103 | 114252 |
| time+time^3^ | -4759 | 9552 |  | -57111 | 114268 |
| time^2^+time^3^ | -4759 | 9552 |  | -57129 | 114305 |
|  |  |  |  |  |  |
| Fit statistics calculated on models with fixed slopes and without robust standard errors due to issues of convergence for some transformations when random slopes were expressed. Superscript numbers for time refer to power terms. ^a^Bayesian information criterion. | | | | | |

**Appendix 5 Unadjusted and adjusted linear trajectories of the mean volume of weekly alcohol consumption until the end of follow-up, stratified by sex and T2DM diagnosis**


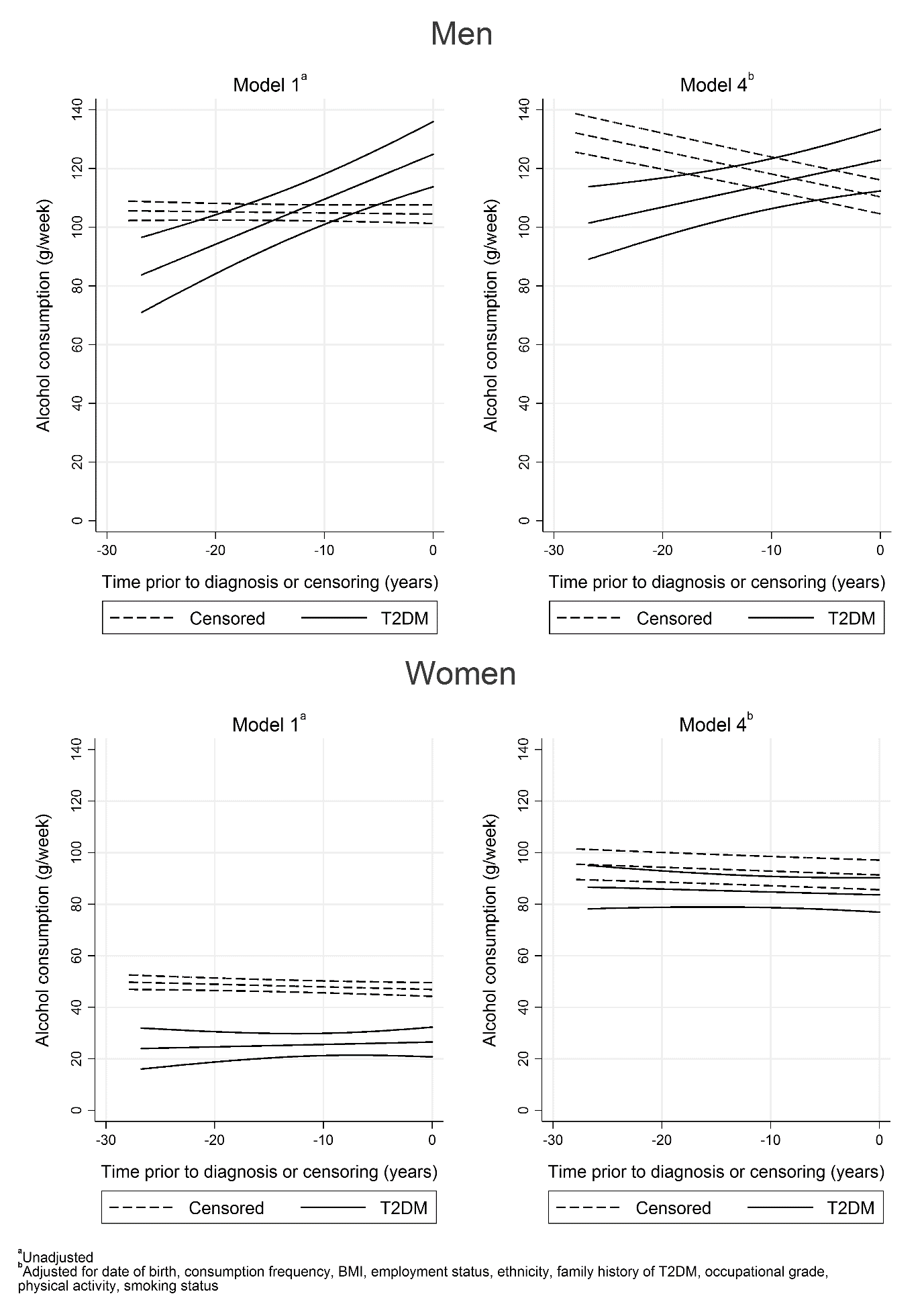


^a^Unadjusted

^b^Adjusted for date of birth, consumption frequency, BMI, employment status, family history of T2DM, occupational grade, physical activity and smoking status. Accordingly, figures are reported according to referent held values (i.e. a non-smoking, physically active, white male).

**Appendix 6 Unadjusted, age-adjusted and multivariable-adjusted linear trajectories of the mean volume of weekly alcohol consumption from baseline until the end of follow-up (excluding non-drinkers), stratified by sex including an interaction with T2DM diagnosis status.**

|  |  |  |  |  |  |  |  |  |  |  |  |
| --- | --- | --- | --- | --- | --- | --- | --- | --- | --- | --- | --- |
|  | **Model 1** |  |  | **Model 2** |  |  | **Model 3** |  |  | **Model 4** |  |
| **Linear random effects models** | **g/week (95% CI)** | **p-value** |  | **g/week (95% CI)** | **p-value** |  | **g/week (95% CI)** | **p-value** |  | **g/week (95% CI)** | **p-value** |
| **Men** |  |  |  |  |  |  |  |  |  |  |  |
| **Consumption volume** |  |  |  |  |  |  |  |  |  |  |  |
| Intercept | 117.5 (114.2, 120.8) | <0.001 |  | 97.6 (91.6, 103.6) | <0.001 |  | 133.1 (127.8, 138.4) | <0.001 |  | 109.3 (103.0, 115.7) | <0.001 |
| Change per 10 years closer to diagnosis or censoring | 0.9 (-0.4, 2.2) | 0.178 |  | 0.9 (-0.4, 2.3) | 0.168 |  | -4.1 (-5.3, -2.8) | <0.001 |  | -8.2 (-9.6, -6.7) | <0.001 |
|  |  |  |  |  |  |  |  |  |  |  |  |
| **Difference in consumption at the time of diagnosis or censoring** |  |  |  |  |  |  |  |  |  |  |  |
| Censoring | Reference |  |  | Reference |  |  | Reference |  |  | Reference |  |
| T2DM | 23.9 (11.6, 36.1) | 0.001 |  | 25.1 (12.9, 37.3) | <0.001 |  | 24.0 (13.3, 34.6) | <0.001 |  | 13.3 (2.8, 23.7) | 0.013 |
|  |  |  |  |  |  |  |  |  |  |  |  |
| **Difference in the rate of change by diagnosis or censoring** |  |  |  |  |  |  |  |  |  |  |  |
| Censoring | Reference |  |  | Reference |  |  | Reference |  |  | Reference |  |
| T2DM | 16.4 (9.3, 23.5) | <0.001 |  | 16.5 (9.4, 23.6) | <0.001 |  | 12.5 (5.9, 19.0) | <0.001 |  | 8.8 (2.3, 15.4) | 0.008 |
|  |  |  |  |  |  |  |  |  |  |  |  |
| **Alcohol consumption frequency** |  |  |  |  |  |  |  |  |  |  |  |
| None in past year | - |  |  | - |  |  | -97.8 (-119.7, -76.0) | <0.001 |  | -101.1 (-108.4, -93.9) | <0.001 |
| <1/week | - |  |  | - |  |  | -102.4 (-105.8, -99.1) | <0.001 |  | -99.5 (-102.9, -96.1) | <0.001 |
| 1-3 times/week | - |  |  | - |  |  | -75.2 (-78.2, -72.2) | <0.001 |  | -73.9 (-76.9, -71.0) | <0.001 |
| Daily or almost daily | - |  |  | - |  |  | Reference |  |  | Reference |  |
|  |  |  |  |  |  |  |  |  |  |  |  |
| *Log-likelihood* | *-125436* |  |  | *-125413* |  |  | *-123813* |  |  | *-123574* |  |
| *Bayesian information criterion* | *250952* |  |  | 250915 |  |  | *247747* |  |  | *247387* |  |
| *Sample size* | *5,327* |  |  | *5,327* |  |  | *5,327* |  |  | *5,327* |  |
|  |  |  |  |  |  |  |  |  |  |  |  |
| **Women** |  |  |  |  |  |  |  |  |  |  |  |
| **Consumption volume** |  |  |  |  |  |  |  |  |  |  |  |
| Intercept | 62.8 (59.7, 65.9) | <0.001 |  | 52.5 (47.2, 57.9) | <0.001 |  | 94.8 (89.5, 100.1) | <0.001 |  | 92.2 (85.6, 98.9) | <0.001 |
| Change per 10 years closer to diagnosis or censoring | 0.5 (-0.8, 1.8) | 0.440 |  | 0.5 (-0.8, 1.8) | 0.459 |  | -1.0 (-2.3, 0.2) | 0.092 |  | -1.4 (-2.7, -0.2) | 0.022 |
|  |  |  |  |  |  |  |  |  |  |  |  |
| **Difference in consumption at the time of diagnosis or censoring** |  |  |  |  |  |  |  |  |  |  |  |
| Censoring | Reference |  |  | Reference |  |  |  |  |  | Reference |  |
| T2DM | -13.3 (-22.3, -4.3) | 0.004 |  | -11.5 (-20.5, -2.5) | 0.013 |  | -6.3 (-13.1, 0.6) | 0.072 |  | -6.8 (-13.9, 0.3) | 0.061 |
|  |  |  |  |  |  |  |  |  |  |  |  |
| **Difference in the rate of change by diagnosis or censoring** |  |  |  |  |  |  |  |  |  |  |  |
| Censoring | Reference |  |  | Reference |  |  |  |  |  | Reference |  |
| T2DM | 3.7 (-2.0, 9.5) | 0.204 |  | 4.0 (-1.8, 9.7) | 0.174 |  | 0.6 (-4.0, 5.3) | 0.796 |  | 0.0 (-4.7, 4.8) | 0.989 |
|  |  |  |  |  |  |  |  |  |  |  |  |
| **Alcohol consumption frequency** |  |  |  |  |  |  |  |  |  |  |  |
| None in past year | - |  |  | - |  |  | -47.8 (-109.4, 13.8) | 0.128 |  | -43.7 (-93.9, 6.6) | 0.088 |
| <1/week | - |  |  | - |  |  | -72.5 (-76.4, -68.6) | <0.001 |  | -70.3 (-74.5, -66.2) | <0.001 |
| 1-3 times/week | - |  |  | - |  |  | -53.2 (-56.7, -49.7) | <0.001 |  | -52.1 (-55.6, -48.6) | <0.001 |
| Daily or almost daily | - |  |  | - |  |  | Reference |  |  | Reference |  |
|  |  |  |  |  |  |  |  |  |  |  |  |
| *Log-likelihood* | *-38702* |  |  | *-38693* |  |  | *-37883* |  |  | *-37823* |  |
| *Bayesian information criterion* | *77476* |  |  | *77466* |  |  | *75872* |  |  | *75860* |  |
| *Sample size* | *2,110* |  |  | *2,110* |  |  | *2,110* |  |  | *2,110* |  |
|  |  |  |  |  |  |  |  |  |  |  |  |
| Model 1: unadjusted; Model 2: as Model 1, plus adjustment for date of birth; Model 3: as Model 2, plus adjustment for consumption frequency; Model 4: as Model 3, plus adjustment for BMI, employment status, ethnicity, family history of T2DM, occupational grade, physical activity, and smoking status. | | | | | | | | | | | |

**Appendix 7 Goodness of fit statistics for linear and non-linear trajectories of alcohol consumption up to and beyond the date of diagnosis, stratified by sex.**

|  | **Men** | |  | **Women** | |
| --- | --- | --- | --- | --- | --- |
| **Function** | **Log-likelihood** | **^a^BIC** |  | **Log-likelihood** | **^a^BIC** |
| **Up to diagnosis** |  |  |  |  |  |
| time^-2^ | -12665 | 25361 |  | -4758 | 9543 |
| time^-1^ | -12665 | 25361 |  | -4758 | 9543 |
| time^1^ | -12648 | 25327 |  | -4759 | 9545 |
| time^2^ | -12647 | 25324 |  | -4759 | 9545 |
| time^3^ | -12649 | 25329 |  | -4759 | 9545 |
| time^-2^+time^-1^ | -12665 | 25368 |  | -4758 | 9549 |
| time^-2^+time | -12648 | 25334 |  | -4758 | 9550 |
| time^-2^+time^2^ | -12647 | 25332 |  | -4758 | 9550 |
| time^-2^+time^3^ | -12649 | 25336 |  | -4758 | 9550 |
| time^-1^+time | -12648 | 25334 |  | -4758 | 9549 |
| time^-1^+time^2^ | -12647 | 25332 |  | -4758 | 9549 |
| time^-1^+time^3^ | -12649 | 25336 |  | -4758 | 9549 |
| time+time^2^ | -12647 | 25332 |  | -4759 | 9552 |
| time+time^3^ | -12647 | 25333 |  | -4759 | 9552 |
| time^2^+time^3^ | -12646 | 25331 |  | -4759 | 9552 |
|  |  |  |  |  |  |
| **After diagnosis** |  |  |  |  |  |
| time^-2^ | -6777 | 13582 |  | -2721 | 5468 |
| time^-1^ | -6775 | 13578 |  | -2721 | 5467 |
| time^1^ | -6767 | 13562 |  | -2719 | 5463 |
| time^2^ | -6766 | 13561 |  | -2718 | 5461 |
| time^3^ | -6768 | 13565 |  | -2718 | 5462 |
| time^-2^+time^-1^ | -6769 | 13573 |  | -2720 | 5472 |
| time^-2^+time | -6766 | 13566 |  | -2718 | 5469 |
| time^-2^+time^2^ | -6766 | 13568 |  | -2718 | 5468 |
| time^-2^+time^3^ | -6768 | 13571 |  | -2718 | 5467 |
| time^-1^+time | -6766 | 13566 |  | -2718 | 5469 |
| time^-1^+time^2^ | -6766 | 13568 |  | -2718 | 5468 |
| time^-1^+time^3^ | -6768 | 13571 |  | -2718 | 5468 |
| time+time^2^ | -6766 | 13567 |  | -2718 | 5468 |
| time+time^3^ | -6766 | 13568 |  | -2718 | 5468 |
| time^2^+time^3^ | -6766 | 13567 |  | -2718 | 5468 |
| Fit statistics calculated on models with fixed slopes and without robust standard errors due to issues of convergence for some transformations when random slopes were expressed. Superscript numbers for time refer to power terms. ^a^Bayesian information criterion. | | | | | |

**Appendix 8 Multivariable-adjusted linear trajectories of the mean volume of weekly alcohol consumption from baseline until the end of follow-up, stratified by sex and T2DM diagnosis. With and without adjustment for BMI.**

|  |  |  |  |  |  |  | |
| --- | --- | --- | --- | --- | --- | --- | --- |
|  |  | **Model 1** |  |  | **Model 2** |  | |
| **Linear random effects models** |  | **g/week (95% CI)** | **p-value** |  | **g/week (95% CI)** | **p-value** | |
| **Men** |  |  |  |  |  |  | |
| **Consumption volume** |  |  |  |  |  |  | |
| Intercept |  | 110.4 (104.6, 116.2) | <0.001 |  | 113.8 (108.0, 119.6) | <0.001 | |
| Change per 10 years closer to diagnosis or censoring |  | -7.8 (-9.1, -6.5) | <0.001 |  | -5.1 (-6.4, -3.9) | <0.001 | |
|  |  |  |  |  |  |  | |
| **Difference in consumption at the time of diagnosis or censoring** |  |  |  |  |  |  | |
| Censoring |  | Reference |  |  | Reference |  | |
| T2DM |  | 12.5 (3.2, 21.8) | 0.008 |  | 19.9 (10.5, 29.3) | <0.001 | |
|  |  |  |  |  |  |  | |
| **Difference in the rate of change by diagnosis or censoring** |  |  |  |  |  |  | |
| Censoring |  | Reference |  |  | Reference |  | |
| T2DM |  | 8.0 (2.4, 13.6) | 0.005 |  | 10.2 (4.5, 15.8) | <0.001 | |
|  |  |  |  |  |  |  | |
| **Alcohol consumption frequency** |  |  |  |  |  |  | |
| None in past year |  | -137.9 (-143.1, -132.7) | <0.001 |  | -138.8 (-143.8, -133.7) | <0.001 | |
| <1/week |  | -113.0 (-116.5, -109.6) | <0.001 |  | -113.7 (-117.2, -110.2) | <0.001 | |
| 1-3 times/week |  | -76.1 (-79.1, -73.1) | <0.001 |  | -76.3 (-79.3, -73.3) | <0.001 | |
| Daily or almost daily |  | Reference |  |  | Reference |  | |
|  |  |  |  |  |  |  | |
| *Log-likelihood* |  | *-139692* |  |  | *-139773* |  | |
| *Bayesian information criterion* |  | *279626* |  |  | *279778* |  | |
| *Sample size* |  | *5,625* |  |  | *5,625* |  | |
|  |  |  |  |  |  |  | |
| **Women** |  |  |  |  |  |  | |
| **Consumption volume** |  |  |  |  |  |  | |
| Intercept |  | 91.4 (85.7, 97.2) | <0.001 |  | 91.9 (86.1, 97.6) | <0.001 | |
| Change per 10 years closer to diagnosis or censoring |  | -1.5 (-2.5, -0.5) | 0.002 |  | -1.0 (-2.0, -0.1) | 0.026 | |
|  |  |  |  |  |  |  | |
| **Difference in consumption at the time of diagnosis or censoring** |  |  |  |  |  |  | |
| Censoring |  | Reference |  |  | Reference |  | |
| T2DM |  | -7.8 (-12.4, -3.1) | 0.001 |  | -6.2 (-10.6, -1.7) | 0.006 | |
|  |  |  |  |  |  |  | |
| **Difference in the rate of change by diagnosis or censoring** |  |  |  |  |  |  | |
| Censoring |  | Reference |  |  | Reference |  | |
| T2DM |  | -1.1 (-4.4, 2.1) | 0.495 |  | -0.8 (-4.0, 2.5) | 0.647 | |
|  |  |  |  |  |  |  | |
| **Alcohol consumption frequency** |  |  |  |  |  |  | |
| None in past year |  | -88.8 (-93.1, -84.5) | <0.001 |  | -88.4 (-92.7, -84.1) | <0.001 | |
| <1/week |  | -79.9 (-83.7, -76.1) | <0.001 |  | -79.6 (-83.3, -75.8) | <0.001 | |
| 1-3 times/week |  | -53.9 (-57.4, -50.4) | <0.001 |  | -53.7 (-57.2, -50.2) | <0.001 | |
| Daily or almost daily |  | Reference |  |  | Reference |  | |
|  |  |  |  |  |  |  | |
| *Log-likelihood* |  | *-50854* |  |  | *-50859* |  | |
| *Bayesian information criterion* |  | *101929* |  |  | *101930* |  | |
| *Sample size* |  | *2,492* |  |  | *2,492* |  | |
|  |  |  |  |  |  |  | |
| Model 1: Adjusted for date of birth, consumption frequency, BMI, employment status, ethnicity, family history of T2DM, occupational grade, physical activity, and smoking status; Model 2: As Model 1 minus adjustment for BMI. | | | | | | |  |

**Appendix 9 Multivariable-adjusted linear trajectories of the mean volume of weekly alcohol consumption from baseline until the end of follow-up, stratified by sex and T2DM diagnosis. All participants versus those with ≥3 person-observations each.**

|  | **Model 1^a^** | |  | **Model 2^b^** | |
| --- | --- | --- | --- | --- | --- |
| **Linear random effects models** | **g/week (95% CI)** | **p-value** |  | **g/week (95% CI)** | **p-value** |
| **Men** |  |  |  |  |  |
| **Consumption volume** |  |  |  |  |  |
| Intercept | 110.4 (104.6, 116.2) | <0.001 |  | 110.2 (104.2, 116.1) | <0.001 |
| Change per 10 years closer to diagnosis or censoring | -7.8 (-9.1, -6.5) | <0.001 |  | -7.6 (-9.0, -6.3) | <0.001 |
|  |  |  |  |  |  |
| **Difference in consumption at the time of diagnosis or censoring** |  |  |  |  |  |
| Censoring | Reference |  |  | Reference |  |
| T2DM | 12.5 (3.2, 21.8) | 0.008 |  | 11.1 (0.9, 21.4) | 0.033 |
|  |  |  |  |  |  |
| **Difference in the rate of change by diagnosis or censoring** |  |  |  |  |  |
| Censoring | Reference |  |  | Reference |  |
| T2DM | 8.0 (2.4, 13.6) | 0.005 |  | 7.7 (1.9, 13.6) | 0.010 |
|  |  |  |  |  |  |
| *Log-likelihood* |  | *-139692* |  |  | *-133998* |
| *Bayesian information criterion* |  | *279626* |  |  | 268237 |
| *Sample size* |  | *5,625* |  |  | *5,040* |
|  |  |  |  |  |  |
| **Women** |  |  |  |  |  |
| **Consumption volume** |  |  |  |  |  |
| Intercept | 91.4 (85.7, 97.2) | <0.001 |  | 90.5 (84.6, 96.4) | <0.001 |
| Change per 10 years closer to diagnosis or censoring | -1.5 (-2.5, -0.5) | 0.002 |  | -1.6 (-2.5, -0.6) | 0.001 |
|  |  |  |  |  |  |
| **Difference in consumption at the time of diagnosis or censoring** |  |  |  |  |  |
| Censoring | Reference |  |  | Reference |  |
| T2DM | -7.8 (-12.4, -3.1) | 0.001 |  | -7.9 (-13.2, -2.6) | 0.003 |
|  |  |  |  |  |  |
| **Difference in the rate of change by diagnosis or censoring** |  |  |  |  |  |
| Censoring | Reference |  |  | Reference |  |
| T2DM | -1.1 (-4.4, 2.1) | 0.495 |  | -0.9 (-4.5, 2.6) | 0.607 |
|  |  |  |  |  |  |
| *Log-likelihood* |  | *-50854* |  |  | *-47807* |
| *Bayesian information criterion* |  | *101929* |  |  | 95833 |
| *Sample size* |  | *2,492* |  |  | *2,135* |
|  |  |  |  |  |  |
| ^a^Adjusted for date of birth, BMI, consumption frequency, employment status, ethnicity, family history of T2DM, occupational grade, physical activity, and smoking status. | | | | | |
| ^b^As Model 1, restricted to participants with ≥3 person-observations. | |  |  |  |  |

**Appendix 10 Baseline characteristics of Whitehall II participants, stratified by sex and number of non-responding phases.**

|  | **Men** | | | |  | **Women** | | | |
| --- | --- | --- | --- | --- | --- | --- | --- | --- | --- |
|  | **0 phases** | **1-3 phases** | **≥4 phases** |  |  | **0 phases** | **1-3 phases** | **≥4 phases** |  |
|  | **% (95% CI) n** | **% (95% CI) n** | **% (95% CI) n** | **P for difference^a^** |  | **% (95% CI) n** | **% (95% CI) n** | **% (95% CI) n** | **P for difference^a^** |
| **Age** |  |  |  |  |  |  |  |  |  |
| Mean years | 44.2 (44.0, 44.4)^b^ 3,919 | 43.5 (43.2, 43.8)^b^ 1,251 | 43.5 (43.1, 43.9)^b^ 810 | <0.001 |  | 44.6 (44.3, 44.9)^b^ 1,555 | 45.9 (45.4, 46.3)^b^ 710 | 46.2 (45.7, 46.6)^b^ 649 | <0.001 |
|  |  |  |  |  |  |  |  |  |  |
| **Alcohol consumption volume** |  |  |  |  |  |  |  |  |  |
| Median g/week | 63.2 (31.6, 134.3)^c^ 3,891 | 71.1 (31.6, 150.1)^c^ 1,239 | 47.4 (15.8, 126.4)^c^ 801 | <0.001 |  | 31.6 (7.9, 63.2)^c^ 1,534 | 23.7 (0.0, 55.3)^c^ 707 | 15.8 (0.0, 47.4)^c^ 641 | <0.001 |
|  |  |  |  |  |  |  |  |  |  |
| **Alcohol consumption frequency** |  |  |  |  |  |  |  |  |  |
| None in past year | 2.2 (1.8, 2.7) 87 | 4.1 (3.1, 5.3) 51 | 4.3 (3.1, 6.0) 35 | <0.001 |  | 5.2 (4.2, 6.4) 80 | 7.6 (5.9, 9.8) 54 | 7.4 (5.6, 9.7) 48 | <0.001 |
| <1/week | 18.8 (17.6, 20.1) 736 | 19.1 (17.0, 21.4) 239 | 25.6 (22.7, 28.7) 206 |  |  | 35.2 (32.9, 37.7) 547 | 41.0 (37.4, 44.7) 290 | 45.4 (41.6, 49.2) 294 |  |
| 1-3 times/week | 44.7 (43.1, 46.2) 1,747 | 42.5 (39.8, 45.3) 531 | 39.5 (36.1, 42.9) 318 |  |  | 36.9 (34.6, 39.4) 573 | 33.8 (30.4, 37.4) 239 | 30.6 (27.1, 34.2) 198 |  |
| Daily or almost daily | 34.3 (32.8, 35.8) 1,341 | 34.3 (31.7, 36.9) 428 | 30.6 (27.6, 33.9) 247 |  |  | 22.7 (20.7, 24.8) 352 | 17.5 (14.9, 20.5) 124 | 16.7 (14.0, 19.7) 108 |  |
|  |  |  |  |  |  |  |  |  |  |
| **BMI** |  |  |  |  |  |  |  |  |  |
| Mean kg/m^2^ | 24.3 (24.2, 24.4)^b^ 3,912 | 24.8 (24.6, 24.9)^b^ 1,250 | 24.6 (24.4, 24.8)^b^ 809 | <0.001 |  | 24.3 (24.1, 24.5)^b^ 1,555 | 24.9 (24.6, 25.3)^b^ 710 | 24.9 (24.6, 25.2)^b^ 649 | <0.001 |
| **Ethnicity** |  |  |  |  |  |  |  |  |  |
| White | 94.9 (94.1, 95.5) 3,716 | 89.1 (87.3, 90.8)  1,108 | 84.3 (81.5, 86.6) 669 | <0.001 |  | 88.8 (87.1, 90.3) 1,379 | 81.6 (78.6, 84.3) 573 | 80.7 (77.4, 83.7) 503 | <0.001 |
| South Asian | 3.7 (3.1, 4.3) 143 | 6.8 (5.6, 8.4) 85 | 9.7 (7.8, 12.0) 77 |  |  | 5.4 (4.4, 6.7) 84 | 8.3 (6.4, 10.5) 58 | 9.1 (7.1, 11.7) 57 |  |
| Other^d^ | 1.5 (1.1, 1.9) 58 | 4.0 (3.1, 5.3) 50 | 6.0 (4.6, 7.9) 48 |  |  | 5.8 (4.7, 7.1) 90 | 10.1 (8.1, 12.6) 71 | 10.1 (8.0, 12.7) 63 |  |
|  |  |  |  |  |  |  |  |  |  |
| **Family history of T2DM** |  |  |  |  |  |  |  |  |  |
| No | 9.9 (9.0, 10.9) 384 | 10.0 (8.4, 11.8) 123 | 11.2 (9.2, 13.6) 89 | 0.536 |  | 13.3 (11.7, 15.1) 203 | 13.3 (11.0, 16.0) 93 | 15.0 (12.4, 18.0) 95 | 0.539 |
| Yes | 90.1 (89.1, 91.0) 3,485 | 90.0 (88.2, 91.6)  1,109 | 88.8 (86.4, 90.8) 704 |  |  | 86.7 (84.9, 88.3)  1,322 | 86.7 (84.0, 89.0) 606 | 85.0 (82.0, 87.6) 537 |  |
|  |  |  |  |  |  |  |  |  |  |
| **Occupational grade** |  |  |  |  |  |  |  |  |  |
| Administrative (top) | 43.1 (41.6, 44.7) 1,689 | 31.7 (29.1, 34.3) 396 | 28.3 (25.3, 31.5) 229 | <0.001 |  | 16.7 (14.9, 18.6) 259 | 7.3 (5.6, 9.5) 52 | 5.4 (3.9, 7.4) 35 | <0.001 |
| Professional (middle) | 52.4 (50.8, 53.9) 2,052 | 55.3 (52.5, 58.1) 692 | 52.8 (49.4, 56.3) 428 |  |  | 46.9 (44.5, 49.4) 730 | 32.3 (28.9, 35.8) 229 | 31.7 (28.3, 35.4) 206 |  |
| Clerical (bottom) | 4.5 (3.9, 5.2) 178 | 13.0 (11.3, 15.0) 163 | 18.9 (16.3, 21.7) 153 |  |  | 36.4 (34.0, 38.8) 566 | 60.4 (56.8, 64.0) 429 | 62.9 (59.1, 66.5) 408 |  |
|  |  |  |  |  |  |  |  |  |  |
| **Physical activity^e^** |  |  |  |  |  |  |  |  |  |
| Inactive | 7.6 (6.8, 8.5) 297 | 10.3 (8.7, 12.1) 126 | 14.1 (11.8, 16.7) 113 | <0.001 |  | 20.3 (18.3, 22.4) 310 | 28.5 (25.2, 32.0) 194 | 29.9 (26.5, 33.6) 188 | <0.001 |
| Below guidelines | 37.9 (36.4, 39.5)  1,476 | 33.9 (31.3, 36.6) 417 | 36.5 (33.3, 39.9) 293 |  |  | 42.5 (40.1, 45.0) 650 | 36.1 (32.6, 39.8) 246 | 36.0 (32.3, 39.8) 226 |  |
| Met guidelines | 54.4 (52.9, 56.0)  2,119 | 55.8 (53.0, 58.6) 686 | 49.4 (45.9, 52.8) 396 |  |  | 37.2 (34.8, 39.6) 568 | 35.4 (31.9, 39.1) 241 | 34.1 (30.5, 37.9) 214 |  |
|  |  |  |  |  |  |  |  |  |  |
| **Smoking** |  |  |  |  |  |  |  |  |  |
| Never | 51.0 (49.4, 52.5)  1,983 | 45.9 (43.2, 48.7) 570 | 46.3 (42.9, 49.8) 371 | <0.001 |  | 59.4 (57.0, 61.9) 919 | 50.8 (47.1, 54.5) 358 | 53.1 (49.2, 56.9) 343 | <0.001 |
| Former | 37.0 (35.5, 38.5) 1,440 | 37.0 (34.3, 39.7) 459 | 33.8 (30.6, 37.2) 271 |  |  | 25.4 (23.2, 27.6) 392 | 24.5 (21.5, 27.9) 173 | 20.0 (17.1, 23.2) 129 |  |
| Current | 12.0 (11.0, 13.1) 467 | 17.1 (15.1, 19.3) 212 | 19.9 (17.2, 22.8) 159 |  |  | 15.2 (13.5, 17.1) 235 | 24.7 (21.6, 28.0) 174 | 26.9 (23.6, 30.5) 174 |  |
| Figures exclude participants who died over the course of the study. Number of participants listed under each variable differs according to item non-response at baseline (phase 1). ^a^To explore differences between non-response groups, one-way ANOVA was used on continuous data, and the chi^2^ test on categorical data (where continuous data exhibited a non-normal distribution, data were log-transformed prior to testing; ^b^Mean and 95% confidence interval; ^c^Median and 25^th^ and 75^th^ percentiles; ^d^e.g. black Caribbean, African and Arabic; ^e^Meeting guidelines (≥150 minutes of moderate-intensity or ≥75 minutes of vigorous-intensity activity per week); inactive (<60 minutes of moderate and <60 minutes of vigorous activity; below guidelines (not inactive or meeting guidelines). | | | | | | | | | |
|  |  |  |  |  |  |  |  |  |  |

**Appendix 11 Baseline characteristics of complete-case Whitehall II participants, stratified by sex and degree of item non-response.**

|  | **Men** | | |  | **Women** | | |
| --- | --- | --- | --- | --- | --- | --- | --- |
|  | **Complete** | **Incomplete** |  |  | **Complete** | **Incomplete** |  |
|  | **% (95% CI) n** | **% (95% CI) n** | **P for difference^a^** |  | **% (95% CI) n** | **% (95% CI) n** | **P for difference^a^** |
| **Age** |  |  |  |  |  |  |  |
| Mean years | 44.1 (43.8, 44.3)^b^ 1,976 | 44.3 (44.1, 44.6)^b^ 1,943 | 0.163 |  | 43.7 (43.3, 44.1)^b^ 697 | 45.3 (44.9, 45.7)^b^ 858 | <0.001 |
|  |  |  |  |  |  |  |  |
| **Alcohol consumption volume** |  |  |  |  |  |  |  |
| Median g/week | 71.1 (31.6, 142.2)^c^ 1,976 | 63.2 (23.7, 126.4)^c^ 1,915 | <0.001 |  | 31.6 (7.9, 71.1)^c^ 697 | 23.7 (0.0, 55.3)^c^ 837 | <0.001 |
|  |  |  |  |  |  |  |  |
| **Alcohol consumption frequency** |  |  |  |  |  |  |  |
| None in past year | 0.8 (0.5, 1.3) 15 | 3.7 (3.0, 4.7) 72 | <0.001 |  | 2.6 (1.6, 4.1) 18 | 7.3 (5.7, 9.2) 62 | <0.001 |
| <1/week | 19.0 (17.4, 20.8) 376 | 18.6 (16.9, 20.4) 360 |  |  | 32.1 (28.8, 35.7) 224 | 37.8 (34.6, 41.1) 323 |  |
| 1-3 times/week | 45.7 (43.6, 48.0) 904 | 43.6 (41.4, 45.8) 843 |  |  | 38.5 (34.9, 42.1) 305 | 35.7 (32.5, 39.0) 305 |  |
| Daily or almost daily | 34.5 (32.4, 36.6) 681 | 34.1 (32.0, 36.3) 660 |  |  | 26.8 (23.7, 30.3) 165 | 19.3 (16.8, 22.1) 165 |  |
|  |  |  |  |  |  |  |  |
| **BMI** |  |  |  |  |  |  |  |
| Mean kg/m^2^ | 24.2 (24.1,24.3)^b^ 1,976 | 24.4 (24.2, 24.5)^b^ 1,936 | 0.051 |  | 23.9 (23.6, 24.2)^b^ 697 | 24.7 (24.4, 25.0)^b^ 858 | <0.001 |
|  |  |  |  |  |  |  |  |
| **Ethnicity** |  |  |  |  |  |  |  |
| White | 96.5 (95.6, 97.2) 1,907 | 93.2 (92.0, 94.2) 1,809 | <0.001 |  | 92.8 (90.7, 94.5) 647 | 85.5 (83.0, 87.7) 732 | <0.001 |
| South Asian | 2.3 (1.7, 3.1) 46 | 5.0 (4.1, 6.1) 97 |  |  | 2.4 (1.5, 3.9) 17 | 7.8 (6.2, 9.8) 67 |  |
| Other^e^ | 1.2 (0.8, 1.7) 23 | 1.8 (1.3, 2.5) 35 |  |  | 4.7 (3.4, 6.6) 33 | 6.7 (5.2, 8.5) 57 |  |
|  |  |  |  |  |  |  |  |
| **Family history of T2DM^d^** |  |  |  |  |  |  |  |
| No | 91.1 (89.8, 92.3) 1,801 | 89.0 (87.5, 90.3) 1,684 | 0.023 |  | 87.9 (85.3, 90.2) 613 | 85.6 (83.1, 87.9) 709 | 0.184 |
| Yes | 8.9 (7.7, 10.2) 175 | 11.0 (9.7, 12.5) 209 |  |  | 12.1 (9.8, 14.7) 84 | 14.4 (12.1, 16.9) 119 |  |
|  |  |  |  |  |  |  |  |
| **Occupational grade** |  |  |  |  |  |  |  |
| Administrative (top) | 43.3 (41.1, 45.5) 856 | 42.9 (40.7, 45.1) 833 | 0.006 |  | 20.5 (17.7, 23.7) 143 | 13.5 (11.4, 16.0) 116 | <0.001 |
| Professional (middle) | 53.2 (51.0, 55.4) 1,051 | 51.5 (49.3, 53.7) 1,001 |  |  | 51.4 (47.6, 55.1) 358 | 43.4 (40.1, 46.7) 372 |  |
| Clerical (bottom) | 3.5 (2.8, 4.4) 69 | 5.6 (4.7, 6.7) 109 |  |  | 28.1 (24.9, 31.6) 196 | 43.1 (39.8, 46.5) 370 |  |
|  |  |  |  |  |  |  |  |
| **Physical activity^e^** |  |  |  |  |  |  |  |
| Inactive | 5.9 (4.9, 7.0) 116 | 9.4 (8.2, 10.8) 181 | <0.001 |  | 17.8 (15.1, 20.8) 124 | 22.4 (19.7, 25.3) 186 | 0.007 |
| Below guidelines | 40.5 (38.4, 42.7) 801 | 35.2 (33.1, 37.4) 675 |  |  | 46.6 (42.9, 50.4) 325 | 39.1 (35.8, 42.5) 325 |  |
| Met guidelines | 53.6 (51.4, 55.8) 1,059 | 55.3 (53.1, 57.5) 1,060 |  |  | 35.6 (32.1, 39.2) 248 | 38.5 (35.3, 41.9) 320 |  |
|  |  |  |  |  |  |  |  |
| **Smoking** |  |  |  |  |  |  |  |
| Never | 50.1 (47.8, 52.3) 989 | 51.9 (49.7, 54.2) 994 | 0.085 |  | 58.2 (54.5, 61.9) 406 | 60.4 (57.1, 63.7) 513 | 0.222 |
| Former | 38.6 (36.5, 40.8) 763 | 35.4 (33.3, 37.5) 677 |  |  | 27.4 (24.2, 30.8) 191 | 23.7 (20.9, 26.7) 201 |  |
| Current | 11.3 (10.0, 12.8) 224 | 12.7 (11.3, 14.3) 243 |  |  | 14.3 (11.9, 17.2) 100 | 15.9 (13.6, 18.5) 135 |  |
|  | | | | | | | |
| Data are restricted to individuals who participated at all phases. Sample sizes differ according to item non-response at baseline (phase 1). ^a^To explore differences between non-response groups, one-way ANOVA was used on continuous data, and the chi^2^ test on categorical data (where continuous data exhibited a non-normal distribution, data were log-transformed prior to testing; ^b^Mean and 95% confidence interval; ^c^Median and 25^th^ and 75^th^ percentiles; ^d^e.g. black Caribbean, African and Arabic; ^e^Meeting guidelines (≥150 minutes of moderate-intensity or ≥75 minutes of vigorous-intensity activity per week); inactive (<60 minutes of moderate and <60 minutes of vigorous activity; below guidelines (not inactive or meeting guidelines). | | | | | | | |
|  |  |  |  |  |  |  |  |

1. Goldberg DP. Detecting Psychiatric Illness by Questionnaire. London: Oxford University Press; 1972. [↑](#endnote-ref-1)
2. Ewing JA. Detecting alcoholism. The CAGE questionnaire. JAMA. 1984; 252(14): 1905–7. [↑](#endnote-ref-2)
3. Folstein MF, Folstein SE, McHugh PR. "Mini-mental state". A practical method for grading the cognitive state of patients for the clinician. J Psychiatr Res. 1975; 12(3): 189–98. [↑](#endnote-ref-3)
4. White IR, Royston P. Imputing missing covariate values for the Cox model. Stat Med. 2009; 28(15): 1982–98. [↑](#endnote-ref-4)
5. Graham JW. Missing Data Analysis: Making it Work in the Real World. Annu Rev Psychol. 2009; 60: 549–576. [↑](#endnote-ref-5)
6. Royston P, White IR. Multiple imputation by chained equations (MICE): implementation in Stata. J Stat Softw. 2011; 45(4): 1–20. [↑](#endnote-ref-6)
